# Replication kinetics of duck enteritis virus UL16 gene in vitro

**DOI:** 10.1186/1743-422X-9-281

**Published:** 2012-11-21

**Authors:** Qin He, Anchun Cheng, Mingshu Wang, Jun Xiang, Dekang Zhu, Yi Zhou, Renyong Jia, Shun Chen, Zhengli Chen, Xiaoyue Chen

**Affiliations:** 1 Institute of Preventive Veterinary Medicine, Sichuan Agricultural University, Wenjiang, Chengdu city, Sichuan, 611130, P.R.China; 2 Avian Disease Research Center, College of Veterinary Medicine of Sichuan Agricultural University, 46# Xinkang Road, Ya’an, Sichuan, 625014, P.R.China; 3 Key Laboratory of Animal Disease and Human Health of Sichuan Province, Sichuan Agricultural University, Wenjiang, Chengdu city, Sichuan, 611130, P.R.China

## Abstract

**Background:**

The function and kinetics of some herpsvirus UL16 gene have been reported. But there was no any report of duck enteritis virus (DEV) UL16 gene.

**Findings:**

The kinetics of DEV UL16 gene was examined in DEV CHv infected duck embryo fibroblasts (DEFs) by establishment of real-time quantitative reverse transcription polymerase chain reaction assay (qRT-PCR) and western-blotting. In this study, UL16 mRNA was transcript at a low level from 0–18 h post-infection (p.i), and peaked at 36 h p.i. It can’t be detected in the presence of acyclovir (ACV). Besides, western-blotting analysis showed that UL16 gene expressed as an apparent 40-KDa in DEV infected cell lysate from 12 h p.i, and rose to peak level at 48 h p.i consistent with the qRT-PCR result.

**Conclusions:**

These results provided the first evidence of the kinetics of DEV UL16 gene. DEV UL16 gene was a late gene and dependent on viral DNA synthesis.

## Findings

Duck enteritis virus (DEV), caused duck viral enteritis (DVE), which was an acute, contagious and widespread disease in the family anatidae of the world, known as Duck plague virus (DPV), is a member of subfamily Alphaherpesvirinae of the family Herpesviridae, and has been assigned into Mardivirus genus [1,2]. DEV is composed of four structures, linear double strand DNA, icosahedral capsid, tegument and envelope. The DEV CHv strain complete genomic sequence and gene library has been constructed in our laboratory [3]. To date, a few of DEV genes of the kinetics has been identified and reported, such as US3 [4], UL31 [5], UL38 [6], UL45 [7], UL51 [8], and UTPase [9]. There was no report of DEV UL16 gene yet. In this study, real-time quantitative reverse transcription polymerase chain reaction assay (qRT-PCR) and western-blotting were used to determine the kinetics of DEV UL16 gene.

**Figure 1:**
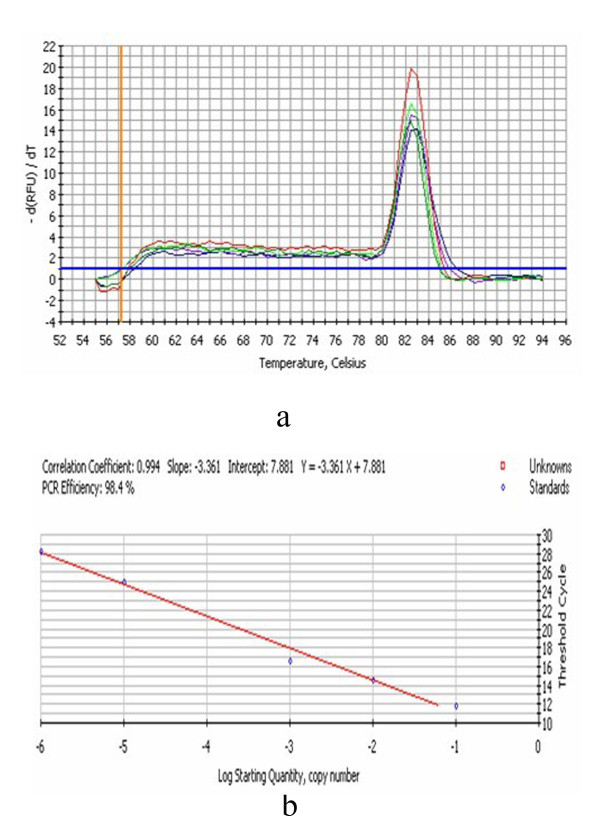
**The qRT-PCR melt curve (a) and standard curve (b) of PMD18-T/UL16.a**: the Tm of amplification fragment was 82.5°C, suggesting that the primers was specific.

**Figure 2:**
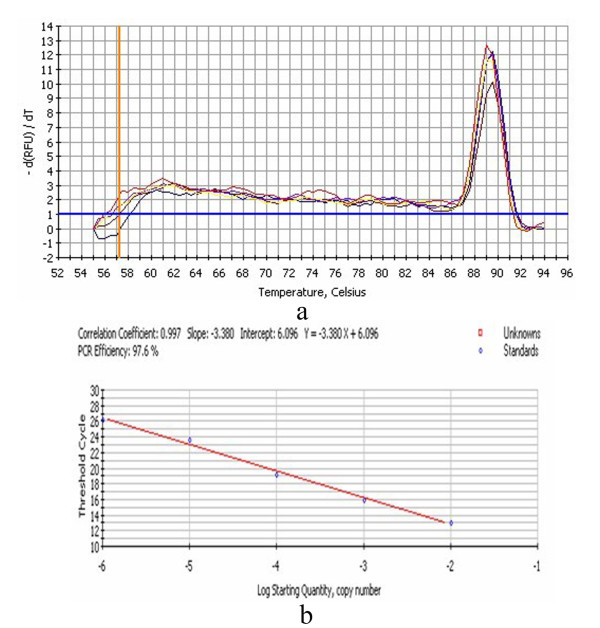
**The qRT-PCR melt curve (a) and standard curve (b) of PMD18-T/β-actin.a**: the Tm of amplification fragment was 89.5°C, suggesting that the primers was specific.

**Figure 3:**
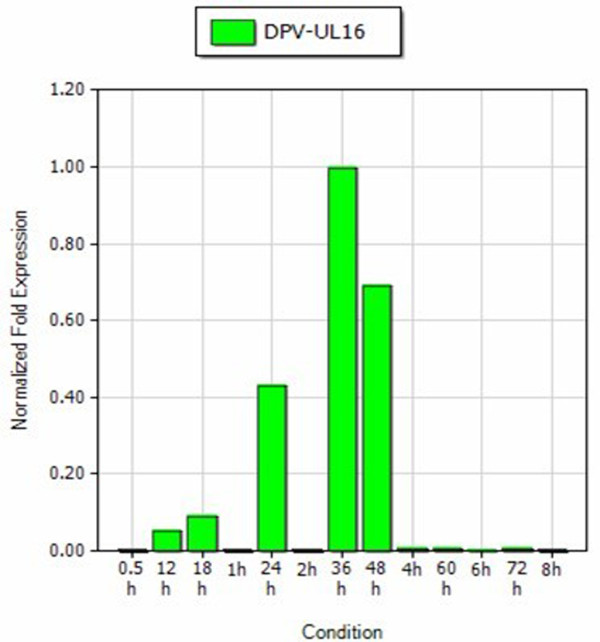
**Kinetics of DEV UL16 gene transcription.**The average relative content of the DEV UL16 gene transcripts was calculated at 0.5, 1, 2, 4, 6, 8, 12, 18, 24, 36, 48, 60 and 72 h p.i. using the 2^-ΔΔCt^ method.

DEV CHv strain was propagated in DEFs. Growth medium consisted of MEM medium (Gibco-BRL) supplemented with 10% calf serum and the maintenance medium supplemented with 2% calf serum. The monolayer DEFs was infected with the DEV CHv. Total cellular RNA was extracted at 0, 0.5, 1, 2, 4, 6, 8, 12, 18, 24, 36, 48, 60 and 72 h p.i, then the RNA was inversed transcribed to cDNA. The primers for qRT-PCR were UL16 (GeneBank Accession No. EU195095) F (5^′^-ATGCCGTGTTTATTGTC-3^′^) and UL16 R (5^′^- GCGGGGTCGTTTCTACTG-3^′^), β-actin F (5^′^- CCGGGCATCGCTGACA-3^′^) and β-actin R (5^′^- GGATTCATCATACTCCTGCTTGCT-3^′^), Standard curves of PMD18-T/UL16 and PMD18-T/β-actin (the recombinant plasmid was constructed in our laboratory) were established (Figure 1 and Figure 2). The transcription kinetics of the DEV UL16 gene in vitro infection was detected by the qRT-PCR as described previous [7]. The qRT-PCR was performed in an 20 μl reaction mixture containing cDNA 2μl, SYBR Green II Mix 10 μl, 1.0 μl primers (10 pmo1/L) respectively, adding ultrapure water to 20 μl. The qRT-PCR consisted of an initial 5 min denaturation step at 95°C, followed by 45 cycles of denaturation (94°C, 30 s), annealing (60°C, 30 s), and extension (72°C, 30 s). Then the fluorescence and melt curve was measured. The relative amount of the UL16 mRNA expression was measured by the method of 2^-ΔΔCt^[10]. As seen in Figure 3, transcription of DEV UL16 mRNA was at low level from 0–18 h, peaked at 36 h, and then reduced after 48 h p.i.

**Figure 4:**
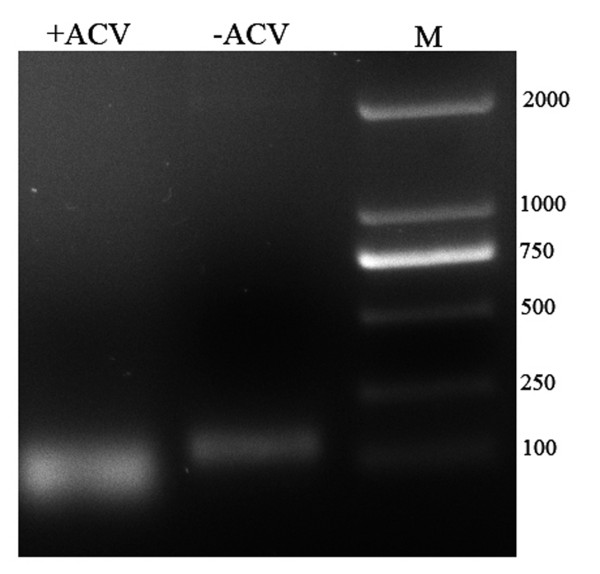
**The dependence of DEV UL16 production on viral DNA synthesis.** The cells were cultured in the presence(+) or absence(−) of 300 mg/ml ACV and the RNA was extracted at 36 h p.i. The RNA was inversed transcribed to cDNA. PCR was performed with cDNA.

To determine the UL16 protein synthesis was highly dependent on viral DNA synthesis, infected DEFs were maintained for 36 h after a 1–2 h adsorption in the presence or absence of ACV (300μg/ml). The RNA was extracted and inversed transcribed to cDNA. The PCR was performed as above. The result showed that the UL16 couldn’t be detected in the presence of ACV (Figure 4), indicating that the UL16 is a true late gene. ACV penetrated infected cells, converting to triphosphate form and then binding to viral deoxyribonucleic acid polymerase, then the viral DNA synthesis was inhibited [11]. As reported in the previous study, no detectable amount of the HSV-2 UL16 protein was produced in the presence of ACV [12]. The same result was obtained when the HSV-1 was in the presence of PAA (a potential inhibitor of viral DNA synthesis) [13]. Hence, we concluded that the DEV UL16 gene may be a true late gene and the UL16 protein synthesis was highly dependent on viral DNA synthesis.

**Figure 5:**
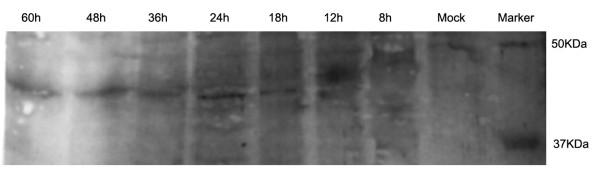
Western-blotting of lysate from mock infected or DEV-infected DEFs with polyclonal antibodies specific to UL16 protein, showing that UL16 is expressed as a 40 kDa protein from 8 h onward following infection.

To confirm the kinetics of DEV UL16 gene expression, DEFs were infected with DEV CHv strain. Cell lysate were prepared at 0, 8, 12, 18, 24, 36, 48, 60 h p.i. The rabbit anti-UL16 polyclonal sera was obtained in the previous study and saved in our laboratory [14]. The DEV UL16 protein was detected using the western-blotting assay. Showing in the Figure 5, an apparent 40KDa protein was detected at 12 h p.i. then increased over time and peaked at 48 h p.i. The qRT-PCR result showed that UL16 mRNA accumulated a maximum at 36 h p.i, but at 48 h p.i in the western-blotting assay. The DNA was transcribed to mRNA, then the mRNA translated to protein. Hence, the highest level of the UL16 protein expression came after the peak of DEV UL16 mRNA. Previous researches had reported that UL16 or homologous-specific transcripts were detected only at late times of infection [15,16], indicating that UL16 is regulated as γ2 gene. UL16 genes are conserved in the whole herpes virus family and the function of UL16 gene may be involved in viral DNA packaging, virion assembly, budding, and egress [12,13,17,18]. The tegument protein of DEV UL51 was first detected at the late stage of infection. Genes can be sorted as immediate early gene (a), early gene (β) and late gene (γ) according to different transcription phase. Universally, structural proteins were encoded by late genes [19]. It could inferred that the DEV UL16 protein may be a structural protein. To confirm this hypothesis, more studies needed to be done.

In this work, we have firstly presented the kinetics of DEV UL16 gene. The productions of DEV UL16 gene accumulated at the late times of infection and couldn’t be detected in the presence of ACV, suggesting that the UL16 gene belonged to γ2 gene and might encode a structural protein which takes part in virion assembly, budding, and egress. The study is in accord with the earlier researches of HSV-1 UL16, HSV-2 UL16 and HCMV UL94 genes. All these results may provide some insights for further studies on the function of DEV UL16 gene.

## Competing interests

The authors declare that they have no competing interests.

## Authors’ contributions

QH carried out most of the experiments and wrote the manuscript. ACC and MSW have critically revised the manuscript and the experimental design. DKZ, YZ, JX, RYJ, CZL and XYC helped in experiments and drafted the manuscript. All authors read and approved the final manuscript.
